# Multiplex Immunoassays Utilizing Differential Affinity Using Aptamers Generated by MARAS

**DOI:** 10.1038/s41598-017-06950-1

**Published:** 2017-07-25

**Authors:** Ji-Ching Lai, Horng-Er Horng, Chin-Yih Hong

**Affiliations:** 1 Research Assistant Center, Chang Hua Show Chwan Health Care System, Changhua, Taiwan; 2 Institute of Electro-optical Science and Technology, National Taiwan Normal University, Taipei, Taiwan; 3 Graduate Institute of Biomedical Engineering, National Chung Hsing University, Taichung, Taiwan

## Abstract

Disease diagnosis typically requires to determine concentration of multiple biomarkers in patient serums. Here, a novel method for multiplex immunoassays is proposed and the feasibility is demonstrated. The method utilizes the differential affinity between aptamers and multiple analytes for multiplex immunoassays. During the selection, aptamers capable of binding to multiple analytes with different affinities are screened from a random oligonucleotide library using the MARAS procedure with different magnetic field conditions for different target analytes. During the detection, the same magnetic field conditions are applied to differentiate different target analytes in blind serums. The results show that the recovery rates of the spiked targets in BD buffer and blind serums are similar. Moreover, there is a minimal interference resulting from non-specific binding of molecules in serums other than the target molecules. Therefore, the use of differential affinities between aptamers and different analytes for multiplex immunoassays is proved to be feasible.

## Introduction

Human diseases such as cancer, autoimmune diseases, and infections are a result of complex processes in which chemicals, cytokines, chemokines, growth factors, adipokines, protein, and nucleic acids are altered. Monitoring changes within these molecules may provide critical information, such as the concentration of specific biomarkers, for early detection, diagnosis, therapy, and prognosis. Using a single biomarker is not sufficient to determine a diagnosis; multiple biomarkers must be used. Candidate biomarkers for diseases are typically identified in the discovery phase of a proteomic screen and many candidate biomarkers have been reported in literature^1^. Detection of multiple analytes has resulted in interest in developing multiplex immunoassays^2^, which have become important due to detection of multiple biomarkers for a wide range of disease diagnosis. Multiplex immunoassays have many advantages, when compared with singleplex immunoassays, which include obtaining faster results^3^, improving throughput^4^, reducing sample volume requirements, and reducing costs and biohazardous waste handling^5–8^.

Today, multiplex immunoassays have been widely used in biomedical research because of the ability to efficiently perform a variety of assay functions in a single reaction vessel, which is generated from a relatively small sample volume. In multiplex immunoassays, high-affinity and high-specificity capture ligands are immobilized in parallel arrays in either planar arrays or on encoded microspheres. After sample incubation, the target analytes are bound to corresponding capture ligands and then form ligand-analyte complexes. After washing to remove unbound substances, reporter ligands conjugated with detection labels attach to complexes. A detection means is used to quantify detection labels, which is then converted to a mass concentration of the target analyte using a pre-determined calibration curve. The number of target analytes that can be analyzed is up to hundreds in a single experiment. Multiplex immunoassays have become powerful analytical tools for biomedical research, however in addition to cost barriers, there are still additional challenges. These include the availability of a large number of highly specific antibodies for a wide range of analytes, cross-reactivity between antibodies, analytes and assay diluents, interference from the matrix effect, the required compromise of assay parameters when developing multiple assays, and the requirement for pre-labeling reporter molecules for detection^9^.

At the clinical level, using multiplex immunoassay requires upfront investment and technical obstacles resulting from the complexity of usage. Fortunately, even though multiplex immunoassays are capable of analyzing a large number of analytes in a single assay, it requires a smaller number of biomarkers for disease diagnosis; no more than four biomarkers^10^. Therefore, there is a demand to develop another type of multiplex immunoassay platform specifically for clinical applications; one that is low cost, user friendly, and time efficient.

In the paper, a novel multiplex immunoassay method specifically suitable for clinical applications is proposed and its feasibility is demonstrated. The technology is based on utilizing differential affinity among capture ligands (aptamers) and corresponding target analytes. This paper includes methods for synthesizing aptamer-based reagents capable of performing multiplex detection in one assay and to detect and quantify multiple analytes in samples that can be highly multiplexed.

## Results and Discussion

### Reverse Validation of Isolated MP Aptamers

**Figure 1:**
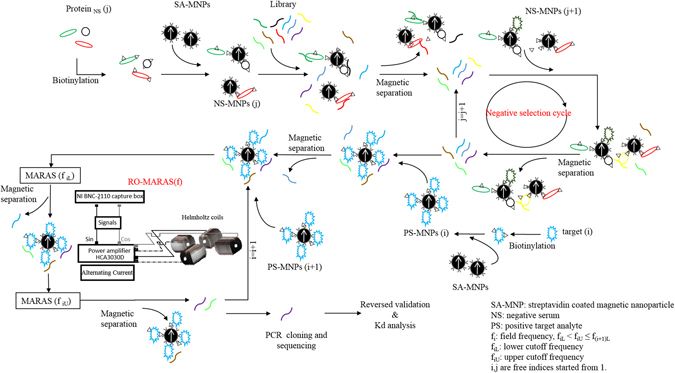
Schematic drawing to illustrate the selection process for aptamers capable of conjugating multiple target analytes having different affinity using the MARAS procedure.

**Figure 2:**
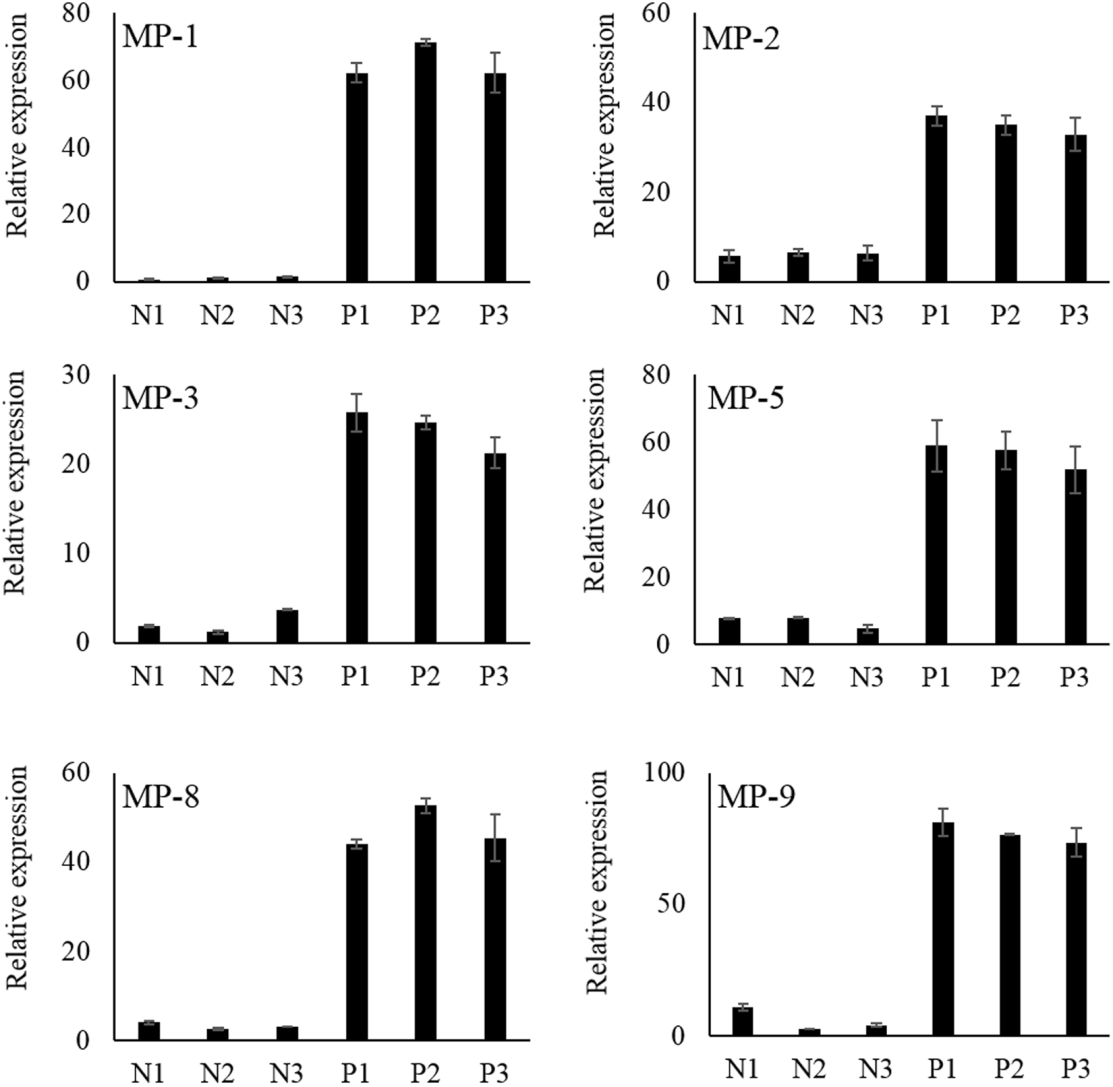
Results of reverse validation of obtained MP-aptamers via q-PCR.

There are six MP-aptamers, 1, 2, 3, 5, 8, and 9 that were isolated using procedure depicted in Fig. 1 and the sequences of the MP-aptamers are listed in Table 1. The validation of the aptamer selection process was performed by reversely targeting positive controls (CRP, HBs Ag, and HCV NS3-MNPs) and negative controls (Negative Serum-1, Negative Serum-2, and Negative Serum-3 MNPs). Results are shown in Fig. 2. The level of binding toward the positive controls were much higher than those of the negative controls. As expected, the MP-aptamer only bound with the target molecules, however not with other molecules in serums. This result demonstrates the promise of the MP-aptamer selection procedure. The MP-1 aptamer was selected and used for the following analyses.

**Table 1:** The sequences of oligonucleotides.

Oligo	5′-sequence-3′
MP-1	CTGCATCACGAAGCCTGGCA
MP-2	AGGTCCTCCGAATGGGACTA
MP-3	CCGGAACACCAGAAGCACGT
MP-5	CCCGTCACCTATTTTTCCGT
MP-8	ACAGGGGAAGAAGCGTCACC
MP-9	CCTTGGCATGATTGTCTCCT

### Dissociation Constants of Isolated MP-1 Aptamer

**Figure 3:**
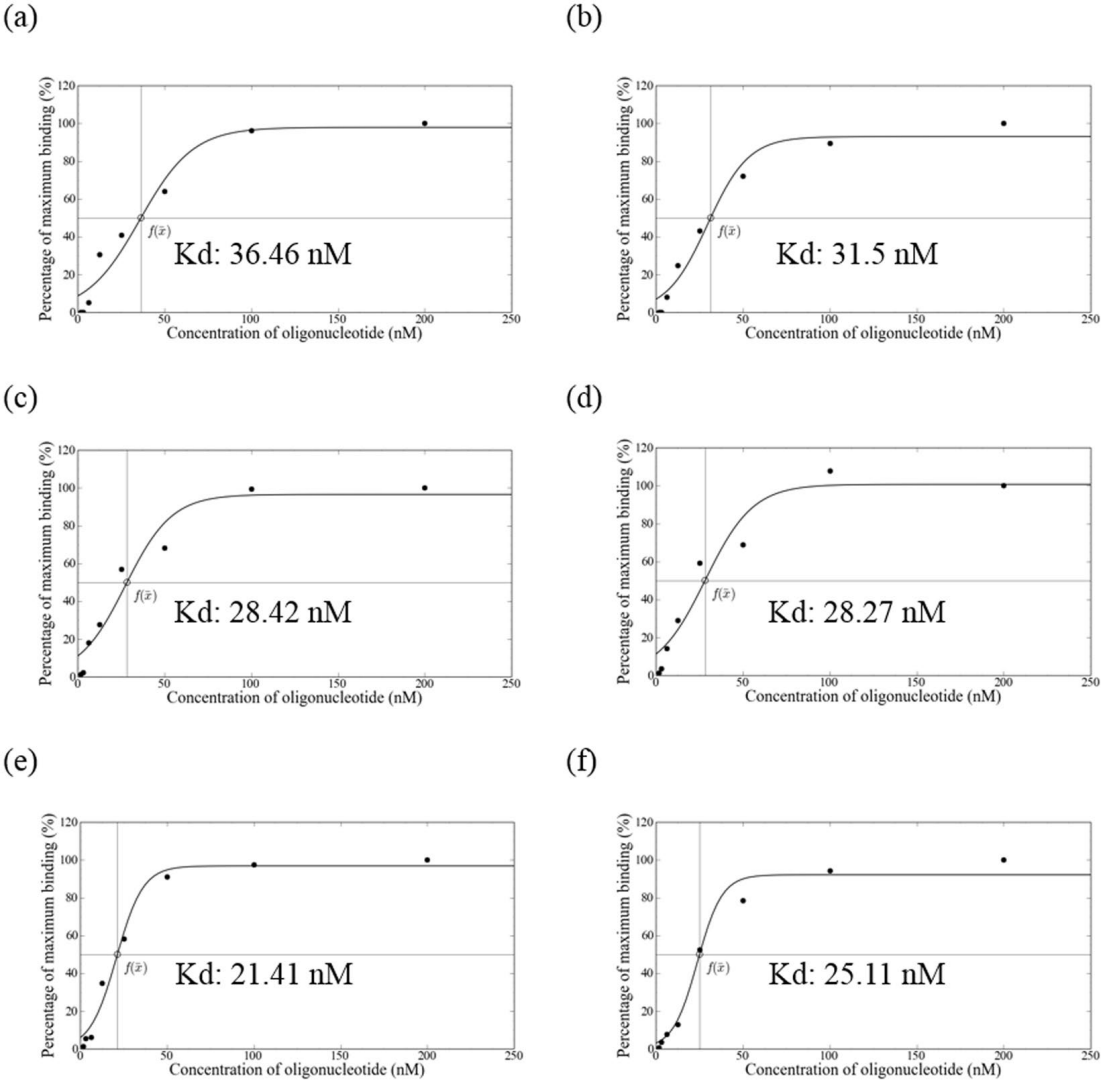
Equivalent dissociation constants of the specific aptamer (MP-1) toward multiple target analytes, (**a,b**) CRP, (**c,d**) HBs Ag, and (**e,f**) HCV NS3 proteins.

As shown previously, the MP-1 aptamer is capable of binding with CRP, HBs Ag, and HCV NS3 proteins. The K_d_ values of the MP-1 aptamer were determined by q-PCR and fitting results through nonlinear regression. The fitting curves and the detail result of dissociation constants are shown in Fig. 3. It shows that the value of K_d_ of the MP-1 aptamer for CRP is 33.98 ± 3.5 nM; for HBs Ag is 28.34 ± 0.1 nM; and, for HCV NS3 is 23.26 ± 2.62 nM. These results indicate the selected MP-1 aptamer is capable of binding with three different targets and can be used for multiplex immunoassays. The values of the dissociation constant of MP-1 aptamer decrease with Target 1, Target 2, and Target 3, sequentially. This result is consistent with our previous results indicating that aptamers generated with the MARAS platform is dependent on the frequency and strength of the magnetic field condition; the higher in which the magnetic field frequency is applied, the lower the dissociation constant obtained^11, 12^. The sequential decrease of the dissociation constant of the MP-1 aptamer for various targets can be attributed to enhancement of competitive mechanisms induced by increasing the magnetic field frequencies at constant field strength for three targets during the screening process.

### Conformation of Multiplex Ability of Isolated MP Aptamers by Aptamer-based ELISA

**Figure 4:**
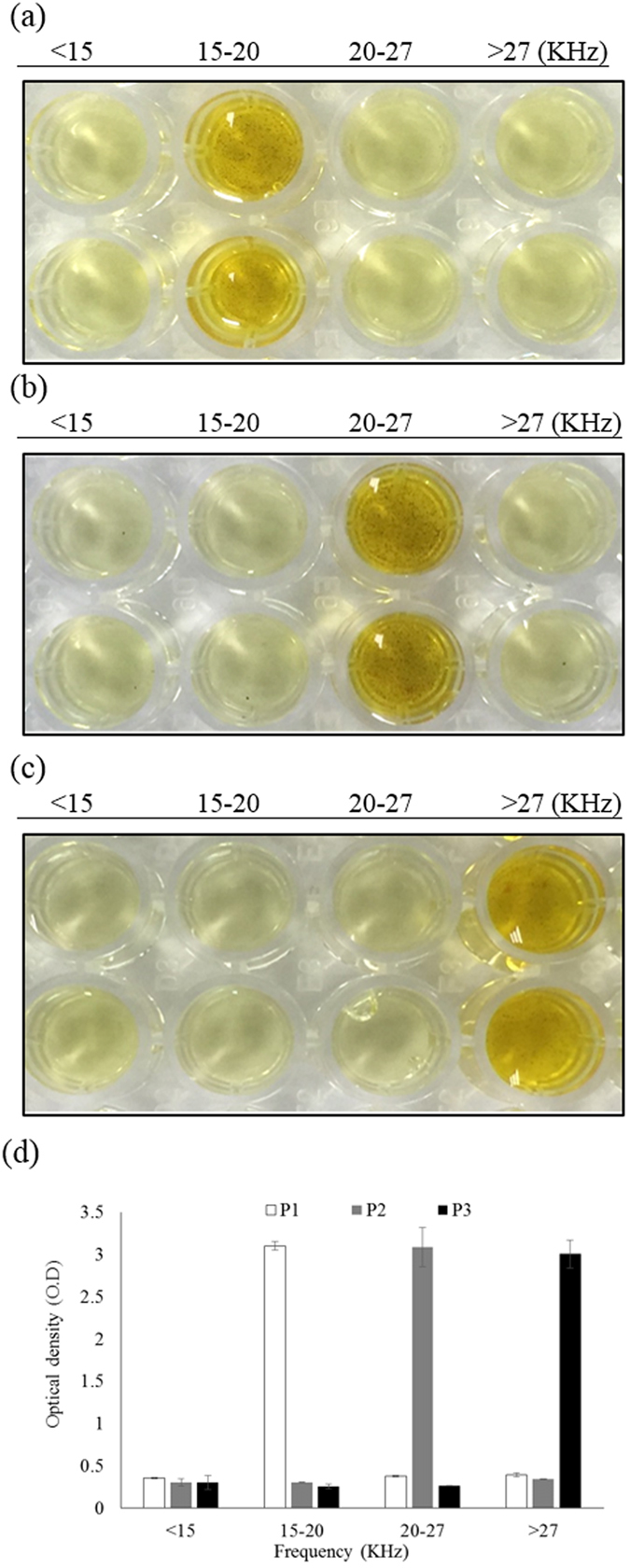
Duplicate photo images (upper and lower rows) for the validation of the MP-1 aptamer capable of conjugating multiple target analytes with different affinities by aptamer-based ELISA, (**a**) CRP (P1), (**b**) HBs Ag (P2), (**c**) HCV NS3 (P3), and (**d**) the measured optical density of control (<15 KHz), (**a**), (**b**), and (**c**).

To verify the multiplex ability of the MP-1 aptamer, an aptamer-based ELISA was performed to verify the binding of MP-1 aptamer toward targets. The detailed experiment was described in the “Method” section below and the results are shown in Fig. 4. Duplicate photo images and the corresponding optical intensity from assaying the CRP, HBs Ag, and HCV NS3 proteins under different magnetic field conditions are also shown. The MP-1 aptamer has the ability to bind with CRP, HBs Ag, and HCV NS3 proteins. The MP-1 aptamer bound with CRP protein, however detached above a magnetic field frequency of 20 KHz and strength of 14 gauss. The MP-1 aptamer bound to HBs Ag protein under a magnetic field strength of 14 gauss and frequency range of up to 27 KHz. Finally, the MP-1 aptamer bound to HCV NS3 protein under a MARAS selection with a frequency of 27 KHz and strength of 14 gauss. Note that the magnetic field conditions used here are identical to those applied during the selection stage. These results indicate that the binding affinity between the MP-1 aptamer and the different targets was dependent on MARAS condition. In other words, it is possible to alter the MARAS condition during the selection stage to obtain aptamers with desired affinities toward targets, which are capable of binding to multiple targets with different affinities. Thus, it is feasible to perform aptamer-based multiplex immunoassays using differential affinities.

### Standard Calibration Curves of the MP-1 Aptamer toward Targets

**Figure 5:**
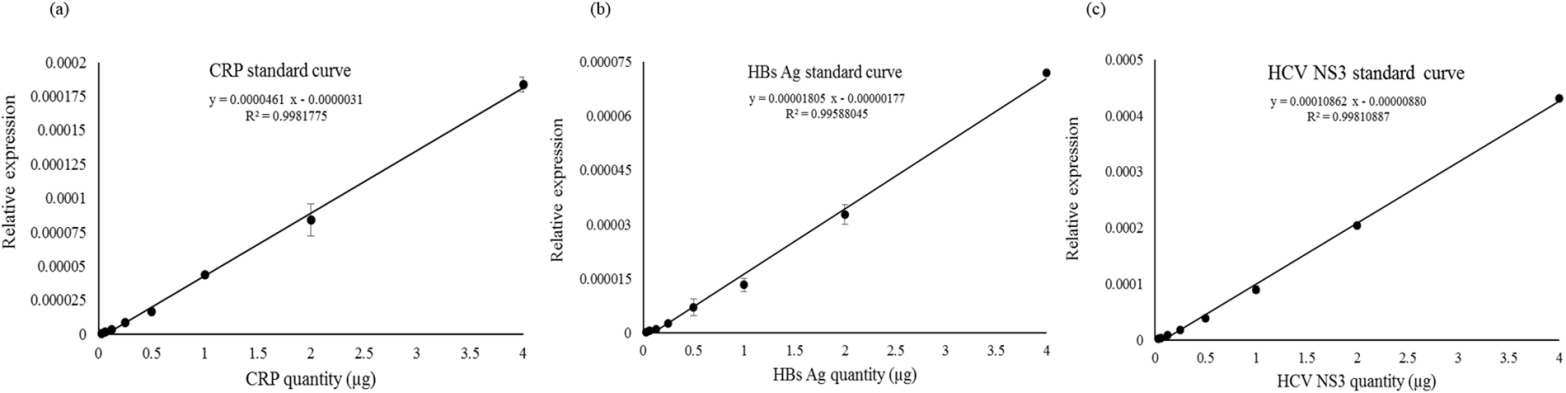
Standard calibration curve for target molecules using the MP-1 aptamer as a capture ligand by q-PCR, the relative expression level vs. (**a**) CRP, (**b**) HBs Ag, and (**c**) HCV NS3 quantities in BD buffer.

A q-PCR process was used to amplify the MP-1 aptamer bound with CRP, HBs Ag, and HCV NS3 proteins, which was described in the “Method” section. A standard calibration curve was determined with a best fit method based on the ct values (expressed as relative expression levels) of a serial dilution of known CRP, HBs Ag, and HCV NS3 samples, respectively. Figure 5 shows the linearly-fitted equation for the calibration curve. As expected, the relative expression levels are linearly proportional to the quantity of the target molecules in samples. The result indicates that dynamic range of measurement is dependent on the amount of MP-aptamer present in the reagent for immunoassays.

### Recovery Rate of Target Analytes in Samples Using MP-1 Aptamer as Capture Ligand

**Figure 6:**
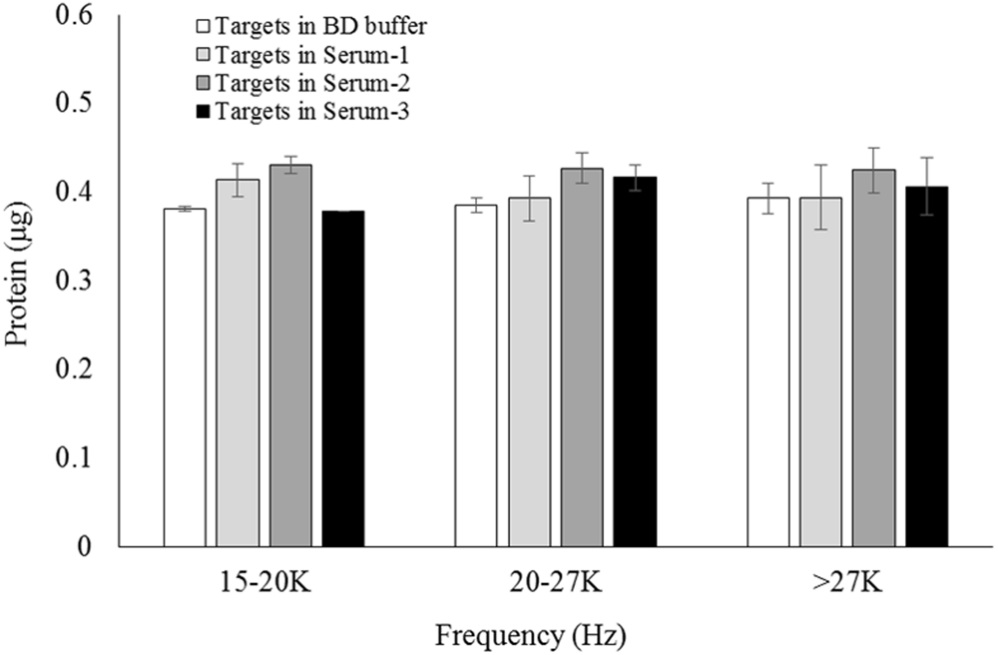
The recovered quantities of spiked target analytes in samples using the MP-1 aptamer as a capture ligand.

Equal amounts (0.4 µg protein) of CRP-MNPs, HBs Ag-MNPs, and HCV NS3-MNPs with or without the blind human serum-MNPs were spiked into BD buffers to form mixed MNP buffer solutions. The mixed MNP buffer solutions were incubated with an excess amount of MP-1 aptamers as described in the “Methods” section. The amount of bound MP-1 aptamer was analyzed by q-PCR, then quantities of CRP, HBs Ag, and HCV NS3 proteins were determined by the relative expression level of the MP-1 aptamer through corresponding standard calibration curves. The recovery rates of target analytes were calculated based on the quantity of spiked target analytes in samples, which was 0.4 µg each ideally, and were between 107.59% and 94.39%. The result is shown in Fig. 6. The protein recovery rate was similar under different magnetic frequency range and there is no significant difference between the measured protein quantities in the BD buffer or Blind Serum-1, -2, and -3. Note that theoretically the levels of recovered target proteins in serums should be equal or higher than that in BD buffer due to nonspecific binding as proteins presented in serums other than target analytes and the deviation can be attributed to the experimental error magnified by q-PCR. On the other hand, by comparing the results with or without the blind human serums, there is limited interference from proteins in the blind serums besides that of other target proteins. This result clearly illustrates that the MP-1 aptamer could specifically bind to Target 1, 2, and 3 in BD buffer and human serums, and can be used for multiplex immunoassay to detect the concentration of CRP, HBs Ag, and HCV NS3 proteins in a single assay.

## Conclusion

This unique multiplex immunoassay technology, which is based on the utilization of differential affinities among capture ligands and corresponding target analytes, is presented and demonstrated in this paper. Aptamers obtained through using the MARAS procedure are capable of binding to multiple analytes with different affinities through altering the magnetic field condition during the aptamer selection process. By applying the same field conditions during the detection process, the quantities of different analytes in the sample can be identified through q-PCR or ELISA. The interference from the non-target molecules in the samples can be minimized by carefully designing the negative selection, for example, using multiple negative selection cycles. These results indicate that if affinity between capture ligands and analytes can be controlled at a desired range, it is possible to synthesize reagents capable of performing multiplex immunoassays utilizing different affinities. This may be particularly applicable for disease diagnosis in clinical applications which there are only the concentrations of a few biomarkers in patient serums needed to be determined.

## Methods

### Oligonucleotide Library and Primer Sequence

The length of the initial oligonucleotide library was 50-mer, which includes a randomized 20-mer midsection (N20) and two primers with 15-mer fixed sections at both ends. The detail sequences of primers used in the study were described in the Supplemental information.

### CRP, HBs Ag, HCV NS3, and Serum Coated Bio-functionalized Magnetic Particles

In this study, human C-reactive proteins (CRP), hepatitis B surface antigens (HBs Ag), and hepatitis C virus non-structural protein 3 (HCV NS3) were purchased from MYBIOSOURCE (MYBIOSOURCE, San Diego USA) and used as binding Target-1 (P1), Target-2 (P2), and Target-3 (P3) respectively, for positive selections. Six healthy volunteers provided serums for three negative selections and three blind tests. The preparation of CRP, HBS Ag, HCV NS3, negative serum-protein and blind serum-protein coated bio-functionalized magnetic particles was according to previous reports^11–13^ and described in the Supplemental information. All experiments, procedures, and methods were carried out in accordance with the IRB approved (CS15071) guidelines and regulations from Chung Shan Medical University Hospital (Taichung, Taiwan). All samples were collected after informed consent of subjects.

### Procedure for Generating Aptamers with Desired Binding Affinity Ranges toward Different Targets

Figure 1 depicts the schematic for generating aptamers having a desired binding affinity ranges toward multiple targets through using the RO-MARAS procedure. The experimental setup of RO-MARAS is identical to that of ref. 11, where the detail working principle was described. A randomized oligonucleotide library was used as the starting library. 100 µM library dissolved in BD buffer (BD: 50 mM NaH_2_PO_4_, pH 8.0, 150 mM NaCl, 5 mM KCl, 2 mM MgCl_2_, 0.05% (v/v) Tween-20) and diluted to 10 μl with BD buffer in a micro-tube. The solution was heated to 95 °C for 5 minutes, snap cooled at 4 °C to form secondary structures, and maintained at room temperature for 30 minutes. First, the negative selection was performed by incubating the library with negative serum-MNP-1 (N1; j), which was obtained through magnetic separation from 5 μl of negative serum-1 MNP reagent, for 30 minutes at room temperature. After magnetic separation, oligonucleotides that bound with negative serum-1 MNPs were removed. The collected supernatant was then incubated with negative serum-MNP-2 (N2; j + 1) and negative serum-3 MNPs (N3; j + 2) and magnetically separated to remove the bound mixture, sequentially. The purpose of the multiple negative selection runs in the designed process was to minimize nonspecific binding and to reduce the possibility of false positives. Theoretically, the more negative selection cycles performed during aptamer selection process, the higher the specificity will be during disease diagnosis for obtained aptamers. The final supernatant was collected for the following positive selection. The CRP-MNPs (P1; i), obtained by magnetic separation from 5 μl of CRP-MNP reagent, was added to the micro-tube containing the final supernatant from the negative selection and incubated for 30 minutes at room temperature. The unbound oligonucleotides were removed with a magnetic stand and the bound mixture was washed twice with 1 ml of BD buffer. 100 µl of BD buffer was added to re-disperse the bound mixture in the micro-tube, which was placed in the RO-MARAS platform. The strength of the rotating magnetic field used through the experiments was 14 gauss. The bound mixture solution was first subjected to a lower-bound rotating magnetic field with a frequency of f_(i)L_ = 15 KHz for 10 minutes at room temperature. To avoid agglomeration from the action of the magnetic field on the magnetic nanoparticles, the bound mixture solution was stirred by pipetting every 2.5 minutes, which was consistent with the following MARAS procedures. A magnetic separation was performed to remove detached oligonucleotides in the supernatant and 100 µl of BD buffer was added to re-disperse the bound mixture. The bound mixture solution was then subjected to MARAS procedure under an upper-bound rotating magnetic field with a frequency of f_(i)U_ = 20 KHz for 10 minutes at room temperature. Another magnetic separation was performed to collect supernatant, which contained aptamers capable of binding with CRP protein selected under the frequency range of 15 KHz ≤ f ≤ 20 KHz. The HBs Ag-MNPs (P2; i + 1) obtained through magnetic separation from 5 μl of HBs Ag-MNP reagent was added to the micro-tube with the collected supernatant and incubated for 30 minutes at room temperature. After removing unbound oligonucleotides with magnetic separation and washing, 100 µl of BD buffer was added to re-disperse the bound mixture. The bound mixture solution was subjected to the next lower-bound rotating magnetic field with a frequency of f_(i+1)L_ = 20 KHz for 10 minutes at room temperature. A magnetic separation was performed to remove detached oligonucleotides in the supernatant and 100 µl of BD buffer was added to re-disperse the bound mixture. The bound mixture solution was subjected to MARAS procedure under the next upper-bound rotating magnetic field with a frequency of f_(i+1)U_ = 27 KHz for 10 minutes at room temperature. A magnetic separation was performed to collect supernatant, which contained aptamers capable of binding with CRP and HBs Ag proteins. These were selected under frequency ranges of 15 KHz ≤ f ≤ 20 KHz and 20 KHz ≤ f ≤ 27 KHz, respectively. The HCV NS3-MNPs (P3; i + 2), obtained through magnetic separation from 5 μl of HCV NS3-MNP reagent, was added into the micro-tube containing collected supernatant and incubated for 30 minutes at room temperature. After a magnetic separation to remove unbound oligonucleotides and washing, 100 µl of BD buffer was added to re-disperse the bound mixture. The bound mixture solution was subjected to MARAS procedure under the final lower-bound rotating magnetic field with a frequency of f_(i+2)L_ = 27 KHz for 10 minutes at room temperature. A magnetic separation was performed to remove supernatant and the bound mixture was retained. The retained bound oligonucleotides in the mixture were eluted from the HCV NS3-MNPs by heating to 95 °C for 5 minutes in 100 µl BD buffer. A magnetic separation was performed to collect the supernatant containing multiplex binding aptamers (MP-aptamer) capable of binding with CRP, HBs Ag, and HCV NS3 proteins. These were selected under frequency ranges of 15 KHz ≤ f ≤ 20 KHz for CRP, 20 KHz ≤ f ≤ 27 KHz for HBs Ag, and 27 KHz ≤ f for HCV NS3. Purification step was then taken to remove HCV NS3 proteins, which were detached from magnetic particles during the heating process, from the supernatant by using a DNA Extraction Miniprep System (Viogene, Taipei, Taiwan) and the obtained aptamers were eluted in 20 μl of ddH_2_O. The subscripts, i and j, used in the field frequency and sample are free indices starting from 1. To assay multiple targets using differential affinities, the frequency of the applied rotating magnetic field must satisfy the condition, f_(i)L_ < f_(i)U_ ≤ f _(i+1)L_. Please note that the upper-bound rotating magnetic field applied for the last round of positive selection was not necessary. Furthermore, the alternating magnetic fields could possibly be used to replace rotating magnetic fields in the procedure using the setup depicted in ref. 12. Moreover, it is worth to be pointed out that any mechanism capable of producing stretch forces (competitive mechanism in refs 11 and 12) on the ligand-analyte bond can be used to develop the platform for multiplex immunoassay utilizing differential affinity, such as mechanical force (for example, hydrodynamic force induced by stringent washing in the case the ligand-analyte complex is attached to a fixed substrate or trapped such as fluidic channels, and centrifugal force induced by spinning the ligand-analyte complex attached to a fixed substrate or trapped such as fluidic channel in a lab-on-a-disc), or electromagnetic force (for example, static magnetic force induced by magnetic gradient field in the case magnetic substances are used and static electric force induced by electric field in the case electrically charged substances are used).

### Cloning, Sequencing, and Motif Analysis of Selected Aptamers

Collected supernatants were subsequently amplified by PCR with Lab-F and Lab-R primers. The PCR reaction, which contained 1.25 U of DNA polymerase (Invitrogen), 0.1 mM of dNTPs, 0.5 mM of MgSO_4_, and 0.5 nM primers, was performed under the following conditions: 5 minutes at 95 °C; 35 cycles of 40 seconds at 95 °C; 40 seconds at 60 °C; 40 seconds at 72 °C; and, 10 minutes at 72 °C. The PCR product was purified by using a DNA Extraction Miniprep System. The purified product was sub-cloned to a pGEM-T Easy vector (Promega, Madison, WI, USA). The cloning procedure was performed according to manufacturer instructions. The plasmids of picked-up colonies were purified by using a High-Speed Plasmid Mini Kit (Geneaid, Taipei, Taiwan) and sequenced using an Applied Biosystems PRISM 3730 DNA automatic sequencer and a Big Dye terminator cycle sequencing kit (Foster City, CA, USA). The secondary structures of the aptamers were predicted by using Mfold^14^.

### Reverse Validation of Selected MP-aptamers

Six aptamers (MP-aptamers) were used to validate the selection process (Fig. 1). For each plasmid selected from the cloning experiments, 10 ng of aptamer clone plasmid was used as a PCR template to generate double strand DNA (dsDNA) of MP-aptamer with Lab-biotin-R and Lab-F primers. The PCR procedure was described in the previous section. After completing PCR amplification, the PCR product was mixed with SA-MNPs obtained through magnetic separation from 5 μl of SA-MNP reagent. The forward single strand MP-aptamer (non-biotinylated strand) was separated from the immobilized complementary strand through incubation with 0.15 N of fresh NaOH for 5 minutes. The SA-MNP bound mixture was removed with a magnetic stand. An equal amount of 0.15 N of HCl was added to the collected supernatant to adjust the final pH to 7.0, after which the forward ssDNA MP-aptamer was precipitated with 1 ml of 100% ice-cold alcohol. The concentration of the single strand MP-aptamer was determined with a NanoDrop 2000c spectrophotometer (Thermo Fisher Scientific, Wilmington, DE, USA). A 100 nM of MP-aptamer in 20 µl of BD buffer was heated to 95 °C for five minutes and cooled at 4 °C to form secondary structures. The MP-aptamer solutions were incubated with CRP-MNPs (P1), HBs Ag-MNPs (P2), HCV NS3-MNPs (P3), and negative serum-MNPs (N1, N2, and N3), individually, for 30 minutes at room temperature, of which the protein MNPs were obtained from 5 µl of corresponding reagents through magnetic separation. A magnetic separation was performed to collect the bound mixture. The collected bound mixture was washed twice with BD buffer and re-dispersed in 20 µl of BD buffer. Another magnetic separation was performed to remove the supernatant and to collect the bound mixture. The bound mixture was re-dispersed in 100 μl of ddH_2_O and heated to 95 °C for 5 minutes to elute aptamers from the MNPs for CRP-MNPs (P1), HBs Ag-MNPs (P2), HCV NS3-MNPs (P3), and negative serum-MNPs (N1, N2, and N3). A magnetic separation was performed to remove the MNPs and to collect the supernatant. The amount of the eluted aptamers in the supernatant was measured using a SYBR-based real-time quantitative PCR (q-PCR). The mixture for each q-PCR run was 10 µl containing 2 μl of nucleic acids, 2.5 µl of SYBR Green PCR master mix (Applied Biosystems) and 0.5 nM of primers. The reaction condition was as follows: 95 °C for 3 minutes; 40 cycles at 94 °C for 30 seconds; 60 °C for 30 seconds; and, 72 °C for 30 seconds. The primers, Lab-F and Lab-R, were used for q-PCR to amplify the nucleic acids.

### Determination of Equilibrium Dissociation Constants of Selected Aptamers by Real-Time Quantitative PCR

The affinity of MP-aptamers toward the CRP, HBs Ag, and HCV NS3 targets was described by the equilibrium dissociation constant (K_d_), which was measured by q-PCR^11–13, 15^. The single strand MP-aptamers were generated and described in the previous sections. A series of progressively diluted MP-aptamers (200 nM to 1.5625 nM) in 20 µl of BD buffer were heated to 95 °C for 5 minutes and cooled at 4 °C to form secondary structures. Partially diluted MP-aptamers were retained as input control (input). CRP-MNPs (P1), HBs Ag-MNPs (P2), and HCV NS3-MNPs (P3) were obtained from 5 μl of CRP-MNP (P1), HBs Ag-MNP (P2), and HCV NS3-MNP (P3) reagents using magnetic stand, respectively. These were then added to each corresponding micro-tube containing diluted MP-aptamers and incubated for 30 minutes at room temperature. Magnetic separation was performed to collect the bound mixture, which was then washed twice with 100 µl of BD buffer. The bound MP-aptamers were eluted from target MNPs by heating the aptamers at 94 °C for 10 minutes in 20 µl of ddH_2_O. The target MNPs in the solution were removed with a magnetic stand and the supernatants were collected. Both the input control and eluted MP-aptamers were precipitated with 1 ml of 100% ice-cold alcohol. The input control and eluted MP-aptamers were individually dissolved in test tubes filled with 100 μl of ddH_2_O. The quantities of MP-aptamers in each test tube, including input control tube and eluted MP-aptamer tubes, were calculated using a q-PCR method. The equilibrium dissociation constant (K_d_) of selected aptamers for the target was measured by q-PCR^11, 13^. The detail q-PCR process was described in Supplemental information.

### Verification of Aptamer Binding by Enzyme-linked Immunosorbent Assay

An aptamer-based ELISA was performed to verify binding of the MP-aptamer. Biotinylated MP-aptamer was synthesized and purchased from MDBio (MDBio, Taipei, Taiwan). 10 nM biotinylated MP-aptamers in each micro-tube containing 20 µl of BD buffer were heated to 95 °C for 5 minutes and cooled at 4 °C for the formation of secondary structures. CRP-MNPs (P1), HBs Ag-MNPs (P2), HCV NS3-MNPs (P3), obtained from 5 μl of corresponding reagent by magnetic separation, were incubated with 10 nM biotinylated MP-aptamer in micro-tubes for 30 minutes at room temperature, separately. After performing magnetic separation to remove unbound aptamers and washing, 100 µl of BD buffer was added to re-disperse the bound mixture. The bound mixture solution was subjected to a rotating magnetic field frequency of 15 KHz for 10 minutes at room temperature. A magnetic separation was performed to remove detached aptamers in the supernatant and labeled “<15 KHz”. 100 µl BD buffer was added to re-disperse the bound mixture. The same process was performed at 20 KHz and 27 KHz, sequentially. All supernatants containing detached corresponding aptamer fractions were collected and labeled “15–20 KHz” and “20–27 KHz”. 100 µl of BD buffer was added to re-disperse the final bound mixture and heated to 95 °C for 5 minutes to elute the aptamers from target MNPs. A magnetic separation was performed to collected the supernatant and labeled “>27 KHz”. Each set of collected supernatants included “<15 KHz”, “15–20 KHz”, “20–27 KHz”, and “>27 KHz” fractions for all three targets. The collected supernatants were incubated with 100 μl of streptavidin-HRP (Sigma-Aldrich, Missouri, USA) in micro-tubes for one hour at room temperature. CRP-MNPs (P1), HBs Ag-MNPs (P2), and HCV NS3-MNPs (P3), obtained from 1 μl of CRP-MNPs, HBs Ag-MNPs, and HCV NS3-MNPs reagents by magnetic separation respectively, were added to the three sets of micro-tubes separately, and incubated for one hour at room temperature. The mixture in the micro-tube was magnetically separated and washed 3 times with 200 μl of BD buffer. The color was developed by adding 100 μl of 3,3′,5,5′-tetramethyl-benzidine (TMB, Sigma-Aldrich) substrate solution and maintaining the mixture at room temperature for 5 minutes. The reaction was terminated with the addition of 100 μl of 2 N HCl and the absorbency was measured in duplicate at 405 nm by using an EMax precision microplate reader (Molecular Devices, CA, USA).

### Establishment of Standard Curve by Real-Time Quantitative PCR

Standard calibration curves were individually determined using a serial dilution of CRP-MNPs, HBs Ag-MNPs, and HCV NS3-MNPs, obtained from 1 μl of CRP-MNP (Target 1), HBs Ag-MNP (Target 2), and HCV NS3-MNP (Target 3) reagents by magnetic separation, respectively, in 10 μl BD buffer. The corresponding target quantities in the diluted solution were reduced by half, starting at 4000, then to 2000, then to 1000 ng, and so on. The final quantity was 31.25 ng. 1 μM MP-aptamer dispersed in 10 μl BD buffer was heated to 95 °C for 5 minutes and cooled at 4 °C for the formation of secondary structures. Target MNPs (Target 1, 2, and 3), obtained from each diluted solution by magnetic separation, was added to each BD buffer solution containing MP-aptamer and incubated for 30 minutes at room temperature. The bound mixture was collected and the supernatant was removed with a magnetic stand. The bound aptamers were eluted from the MNPs by heating at 94 °C for 10 minutes in a final volume with 100 µl of ddH_2_O and the MNPs were removed through magnetic separation. The amount of eluted oligonucleotides in each collected supernatant was analyzed by q-PCR. The q-PCR analyses were performed in duplicate as described in previous sections. The PCR cycle number (expressed as relative expression level), at which the fluorescence intensity reaches a set cycle threshold value (ct), versus target quantities was calculate by 2^−ct^. The standard calibration curve was linearly fitted from sixteen experimental data points. A best fit method was used to calculate the R^2^ value and obtain a linear equation. This standard calibration curve was used in determining the target quantity in samples for the future analyses.

### Determination of Recovery Rate using the MP-Aptamer in Assaying Target-Spiked Human Serums

The three volunteers’ serums were used as blind samples for determining the recovery rate of MP-aptamers as capture ligands, of which CRP, HBs Ag, and HCV NS3 concentrations were undetectable. These were assigned as Blind Serum-1 (B1), Blind Serum-2 (B2), and Blind Serum-3 (B3). The preparation of blind serum MNPs is described in the “Materials” section. A mixture of pure protein of equal quantity (1.6 µg each) of CRP, HBs Ag, and HCV NS3 MNPs, obtained from 0.4 µl of corresponding MNP reagent by magnetic separation, was spiked into 40 µl BD buffer in a micro-tube. One quarter of the mixture solution (10 µl) was used as a control and labeled “Targets in BD buffer”. The remaining mixture solution was equally divided into three parts (10 µl each) in which each part was individually spiked with blind serum MNPs, obtained from 0.1 µl of blind serum MNP reagents through magnetic separation. These were labeled “Targets in Serum-1,” “Targets in Serum-2,” and “Targets in Serum-3” corresponding to Blind Serum-1 (B1), Blind Serum-2 (B2), and Blind Serum-3 (B3), respectively. A 5000 nM of MP-1 aptamer in 20 µl of BD buffer was heated to 95 °C for five minutes and cooled at 4 °C to form secondary structures. The protein MNPs, obtained from the “Targets in BD buffer” by magnetic separation, were added into the micro-tube for 30 minutes at room temperature. The supernatant in the micro-tube was removed with a magnetic stand and the bound mixture was collected and dispersed in 100 µl of BD buffer. The bound mixture solution was placed inside the RO-MARAS platform under an initial RO-MARAS condition with a field frequency of 15 KHz for 10 minutes at room temperature. A magnetic separation was performed to remove the supernatant and 100 µl of BD buffer was added to disperse the retained bound mixture. The bound mixture solution was subjected to a second RO-MARAS condition with a field frequency of 20 KHz for 10 minutes at room temperature. A magnetic separation was performed to collect the supernatant and labeled “15–20 KHz”. 100 µl of BD buffer was added to disperse the retained bound mixture. The bound mixture solution was subjected to a third RO-MARAS condition with a frequency of 27 KHz for 10 minutes at room temperature. A magnetic separation was performed to collect the supernatant and labeled “20–27 KHz”. A 100 µl of BD buffer was added to disperse the retained bound mixture. The bound mixture solution was heated at 94 °C for 10 minutes to elute aptamers from the MNPs. A magnetic separation was performed to collect the supernatant and labeled “>27 KHz”. The amount of aptamers in all the collected supernatants, “15–20 KHz”, “20–27 KHz”, and “>27 KHz”, was analyzed with q-PCR and calculated with the corresponding linear equation of standard calibration curves to determine the CRP, HBs Ag, and HCV NS3 quantities in “Targets in BD buffer” sample. The same procedure was repeated for “Targets in Serum-1”, “Targets in Serum-2”, and “Targets in Serum-3”. The technological foundation of applying the magnetic fields with frequencies of 15 KHz, 20 KHz, and 27 KHz sequentially to differentiate the binding affinity of the aptamer toward target 1, 2, and 3 is outlined below. Firstly, a magnetic field with frequency of 15 KHz was performed to remove nonspecific binding in the bound mixture. Secondly, by applying a magnetic field (20 KHz) which is the upper bound of the magnetic field condition (15~20 KHz) used for target 1 during selection stage, the aptamers bound with the target 1 were detached from bound mixtures and then used as a reporter for target 1 concentration measurement. The retained bound complex still contained aptamers bound to target-2 and -3. Thirdly, the retained bound complex was subjected to a magnetic field (27 KHz) which is the upper bound of magnetic field condition (20~27 KHz) used for target 2 during selection stage. The aptamers bound with target 2 were detached. The detached aptamers were collected and used for calculating the concentration of target 2. Finally, the retained bound mixture contained aptamers bound to target 3. The eluted aptamers from aptamer-target 3 complex were used for estimating the concentration of target 3.

## Electronic supplementary material

Supplementary Information
