# Supplementary material for: Multiplex Immunoassays Utilizing Differential Affinity Using Aptamers Generated by MARAS
Source: Sci Rep. 2017 Jul 25;7:6397. doi: 10.1038/s41598-017-06950-1 (PMC5527020; doi:10.1038/s41598-017-06950-1)
Supplement: Supplementary file 1 — Supplementary Information [file 41598_2017_6950_MOESM1_ESM.pdf]

Supplementary Information

Multiplex Immunoassays Utilizing Differential Affinity Using Aptamers Generated by MARAS

Ji-Ching Lai[a], Horng-Er Horng[b], Chin-Yih Hong[c*]

[a]Research Assistant Center, Chang Hua Show Chwan Health Care System, Changhua, Taiwan.

[b]Institute of Electro-optical Science and Technology, National Taiwan Normal University, Taipei, Taiwan

[c]Graduate Institute of Biomedical Engineering, National Chung Hsing University, Taichung, Taiwan

[*]Correspondence: Chin-Yih Hong

E-mail: cyhong@dragon.nchu.edu.tw

Tel: 886-4-22840733

Fax: 886-4-22852422

Material and Methods

Oligonucleotide Library and Primer Sequence

The oligonucleotide sequence was (5'-AGCAGCACAGAGGTC-N20-GCGTGCTACCGTGAA-3') synthesized and PAGE purified by MDBio (MDBio, Taipei, Taiwan). A set of primers (Lab-F: 5'-AGCAGCACAGAGGTC-3' and the Lab-R: 5'-TTCACGGTAGCACGC-3'), was used to anneal the 5' and 3' degenerating region of the library during PCR amplification. A set of 5'-biotin labeled primers, Lab-biotin-F and Lab-biotin-R, with the above described sequence, was used to isolate the biotin-forward single strand and forward single strand nucleotides from the double strand PCR product, respectively. A universal T7 primer was used to sequence the nucleotide of the selected aptamer (T7: 5'-TAATACGACTCACTATAGGG-3').

CRP, HBs Ag, and HCV NS3 Serum Coated, Bio-functionalized Magnetic Particles

The CRP, HBs Ag, and HCV NS3 protein concentrations for volunteers' serums were undetectable (CRP concentration, <0.02 μg/ml, Beckman DxC analyzer, Beckman Corporation, Fullerton, CA, HBV DNA undetectable, <15 IU/ml, real-time quantitative PCR, HCV DNA undetectable, <15 IU/ml, real-time quantitative PCR). Even though the CRP content was under the detection limit, the CRP content was still present in all serums. To avoid interference during the aptamer selection and validation phases, the CRP content was removed in all serums prior to experiments. 200 μl of individual healthy human serum was incubated with an overdose of latex particles, which consisted of a polystyrene core and a hydrophilic shell covalently bounded with anti-CRP monoclonal antibodies (Siemens Health- care Diagnostics, Eschborn, Germany). CRP present in the serums formed antigen-antibody complexes with latex particles. After centrifugation (10,000 rpm, 5 minutes), CRP present in the serums was removed and the supernatant was collected. Three healthy human serums were assigned Negative Serum-1, Negative Serum-2, and Negative Serum-3 and the other three were labeled Blind Serum-1, Blind Serum-2, and Blind Serum-3. Magnetic nanoparticles were bio-functionalized by coating streptavidin on the outermost surface of magnetic nanoparticles (SA-MNPs) and dispersed in PBS (pH=7.4) to form a SA-MNP reagent, which were purchased from Magqu (Magqu, Taipei, Taiwan). The average hydrodynamic diameter of SA-MNPs in the reagent was 50 nm. The reagent had a concentration of SA-MNPs with 0.3 emu/g. The biotinylation kit (EZ-Link Sulfo-NHS-Biotinylation Kit) was purchased from Pierce (Rockford, IL,

USA). 200 µg of pure CRP, HBs Ag, HCV NS3 proteins (for positive selection), three negative serums (for negative selection), and three blind serums (for blind tests) were biotinylated according to manufacturer instructions. All biotinylated molecules (positive proteins, negative serums, and blind serums) were individually incubated with 50 µl of SA-MNP reagent. The high-affinity binding between the streptavidin and biotin ensured conjugation between the magnetic nanoparticles and biotinylated target molecules (CRP, HBs Ag, and HCV NS3), negative serums (Negative Serums-1, -2, and -3), and blind serums (Blind Serum-1, -2, and -3). The prepared bio-functionalized magnetic nanoparticle reagents included CRP-MNPs (P1), HBs Ag-MNPs (P2), and HCV NS3-MNPs (P3) for positive selection; Negative Serum-1 MNPs (N1), Negative Serum-2 MNPs (N2), and Negative Serum-3 MNPs (N3) for negative selection; and, Blind Serum-1 MNPs (B1), Blind Serum-2 MNPs (B2), and Blind Serum-3 MNPs (B3) for blind tests. If needed, the positive, negative, or blind MNPs were obtained from corresponding positive, negative, or blind reagents through magnetic separation. The collected positive, negative, or blind-MNPs were washed 3 times with BD buffer and collected with a magnetic stand. The preparation of CRP-coated bio-functionalized magnetic nanoparticles (CRP-MNPs) has previously been described in Lai et al[11-13].

MARAS Experimental Setup

The experimental setup and the detailed working principle of RO-MARAS is described in Ref. 11. Here, we briefly describe the experimental setup, which includes a RO-MARAS process using a rotating magnetic field generated by two sets of Helmholtz coils placed orthogonally. Two signals, cos (ωt) and sin (ωt), were fed into a 2-channel power amplifier with a LABVIEW program through an NI BNC-2110 capture box. The two signals were amplified equally, driving coils to produce rotating magnetic fields. Samples were placed at the intersection of the central lines of the two sets of Helmholtz coils and field strength was calibrated using a gauss meter. Due to the application of the rotating magnetic fields during the screening process, this protocol is referred as RO-MARAS[11].

Determination of Equilibrium Dissociation Constants by Real-Time Quantitative PCR

The q-PCR was performed with MicroAmp optical 96-well reaction plates, and the threshold cycle (ct) value was calculated automatically using a maximum correlation coefficient approach with StepOnePlus Real-Time PCR Systems software, version 2.0 (Applied Biosystems). The mixture for each q-PCR run was 10 µl, which contained 2 µl of nucleic acids, 2.5 µl of SYBR Green PCR master mix (Applied Biosystems),

and 0.5 nM of primer Lab forward and Lab reverse. The reaction condition was as follows: 95°C for 3 minutes; 40 cycles at 94°C for 30 seconds; 60°C for 30 seconds; and, 72°C for 30 seconds. The concentrations of the MP-aptamers in the input control and the eluted MP-aptamers were calculated using a 200 nM concentration of MP-aptamers for maximum binding. The K_d value of the selected MP-aptamer was then determined with a saturation binding curve based on the experimental data using the CurveExpert1.3 curve fitting program (curveexpert.webhop.net). The K_d value of the selected MP-aptamer was performed in duplicate for each q-PCR run and expressed as the mean ± standard deviation from the three separate experiments performed.
